# Supplementary material for: Vimentin filaments integrate low-complexity domains in a complex helical structure
Source: Nat Struct Mol Biol. 2024 Apr 17;31(6):939–49. doi: 10.1038/s41594-024-01261-2 (PMC11189308; doi:10.1038/s41594-024-01261-2)
Supplement: Supplementary file 1 — Supplementary Figures 1–10. [file 41594_2024_1261_MOESM1_ESM.pdf]

nature structural & molecular biology

Article https://doi.org/10.1038/s41594-024-01261-2

Vimentin filaments integrate low-complexity domains in a complex helical structure

In the format provided by the authors and unedited

Supplementary Figures

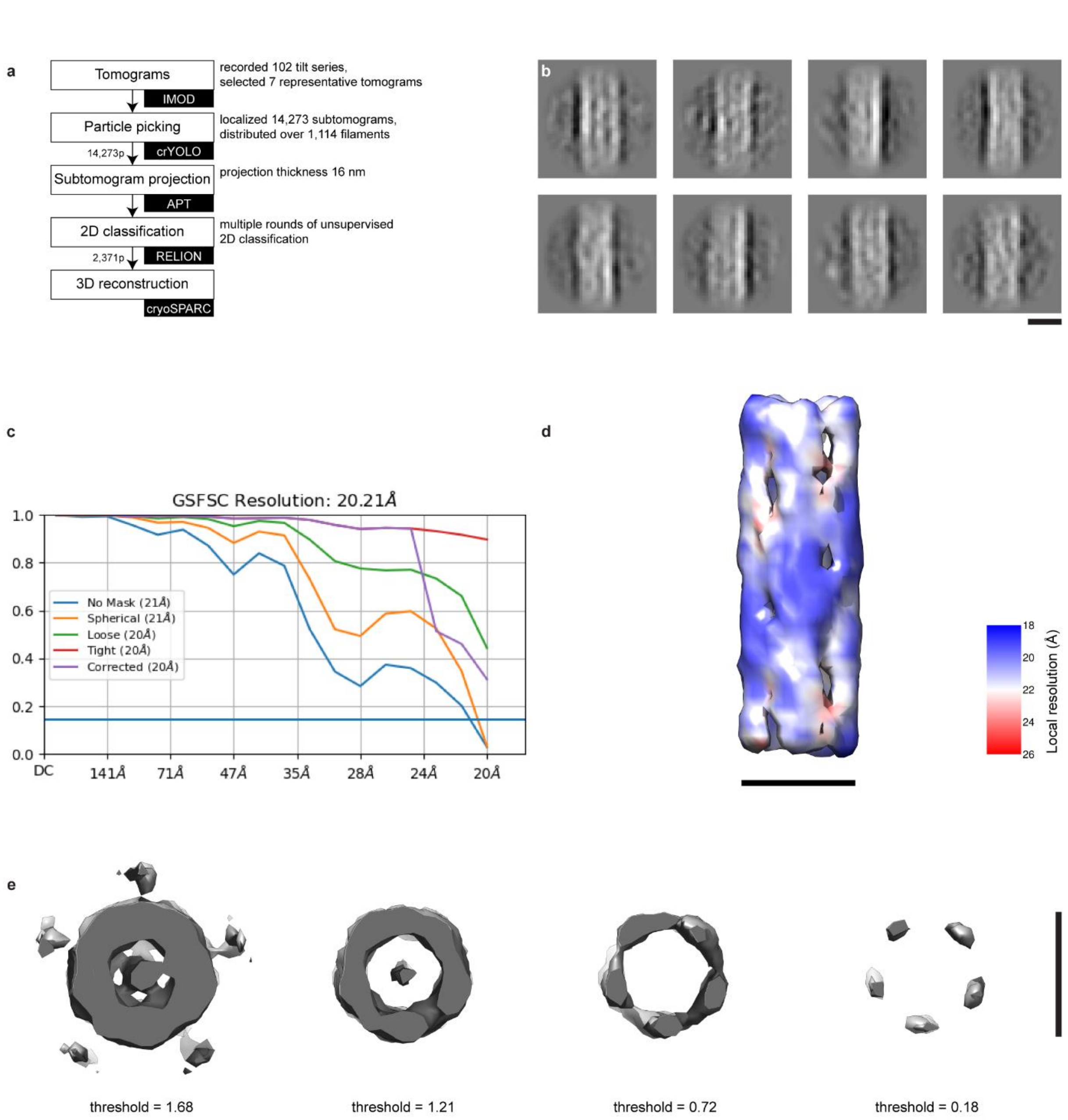

Supplementary Figure 1. Subtomogram averaging of in-situ polymerized VIFs. (**a**) Subtomogram averaging processing workflow. See Methods section for more details. (**b**) Gallery of 2D classes (n=20) calculated from projected subtomograms (n=2371). Scale bar 10 nm. (**c**) FSC resolution measurement of the final VIF subtomogram average with applied helical symmetry. The subtomogram average reached a resolution of 20.2 Å, measured at FSC threshold 0.143. (**d**) Local resolution measurement of this subtomogram average. (**e**) Cross-section views of this subtomogram average at different isosurface thresholds. At high threshold

(~1.68) the connections of the luminal fiber with the filament tube are visible. At low threshold (~0.18) the protofibrils appear as separated units. The isosurface cross-section visualization of this subtomogram average (Fig. 1d, Extended Data Fig. 1d, and Extended Data Fig. 2e) is a montage between two thresholds to allow for counting of the protofibrils (threshold ~0.72) and visualization of the luminal fiber (threshold ~1.21). Scale bar 10 nm.

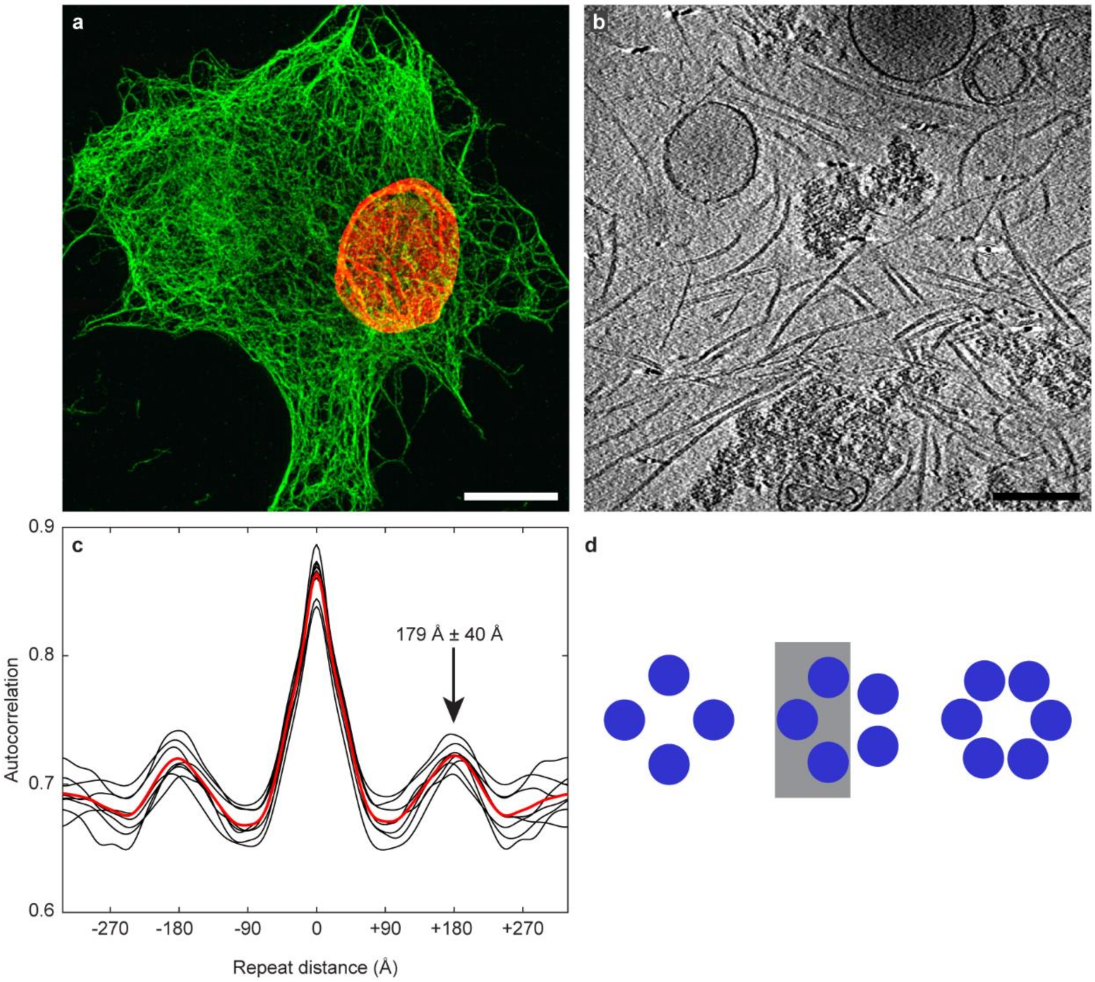

Supplementary Figure 2. Cryo-ET of detergent-treated MEFs. (**a**) Maximum intensity projection of a 3D-SIM image (n=20) of a detergent-treated MEF. The VIF network remains intact following the permeabilization procedure, fixation and staining with vimentin antibody (green). The cell nucleus is stained in red using lamin antibody. Scale bar 10 µm. (**b**) Slice in x-y-direction with 13.76 Å thickness through a cryo-tomogram of detergent-treated MEFs (n=225). Scale bar 200 nm. (**c**) The correlation of each class average (n=8) with itself was calculated and the corresponding autocorrelation profiles along the x-axis were plotted (black lines). The red line is the averaged autocorrelation profile over all class averages. The pattern in the averages repeats at a distance of 179 Å ± 40 Å. (**d**) Model to explain the boundary asymmetry in the class averages. If VIFs are assembled from 5 protofibrils, one side of the VIF would appear brighter in projection (grey rectangle). However, if they are assembled from 4 or 6 protofibrils the VIF boundaries would have similar densities in projection.

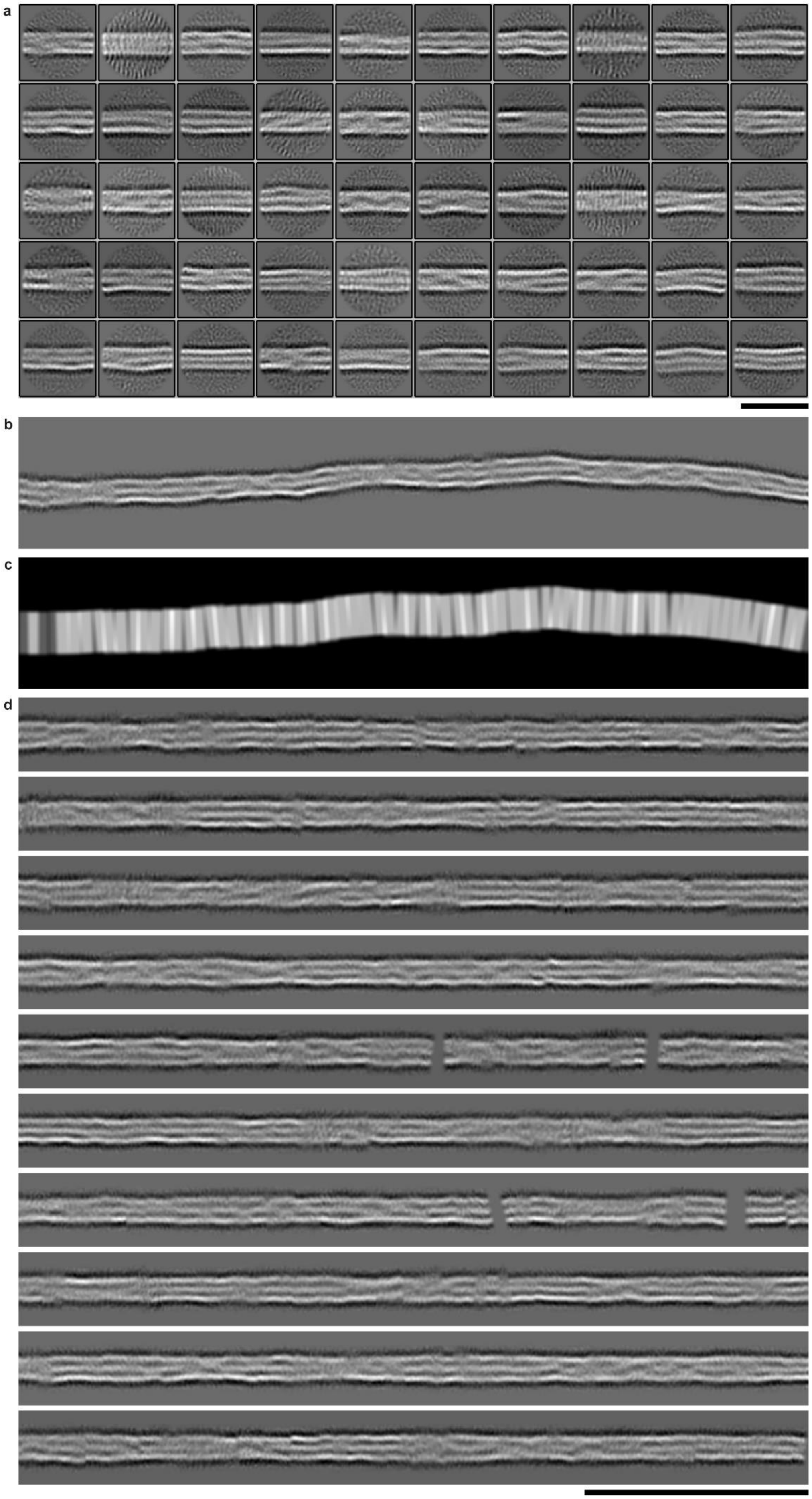
a
b
c
d

Supplementary Figure 3. Computational assembly of VIFs. (**a**) VIF segments (615,106 particles with a size of 38 x 38 nm^2) were extracted from tomograms of detergent-treated MEFs and combined with 2D classification. The picking distance between the segments was set to 55 Å and the projection thickness to 220 Å [1]. The displayed class averages were used for subsequent computational filament assembly. Scale bar 35 nm. (**b**) The computationally assembled VIFs (ca-VIFs) allow to follow the progression of the filaments with improved signal-to-noise ratio over a substantial length (n=5205). The displayed filament box is 353 nm wide. (**c**) The ca-VIFs are represented by a series of transformed, tailored, and overlapping class averages. The densities are normalized according to the local overlap of the class averages. The image shows the normalization mask that was applied to the ca-VIF shown above. More overlap between the class averages is indicated by brighter regions. (**d**) Gallery of unbent ca-VIFs (n=5205). Scale bar 100 nm.

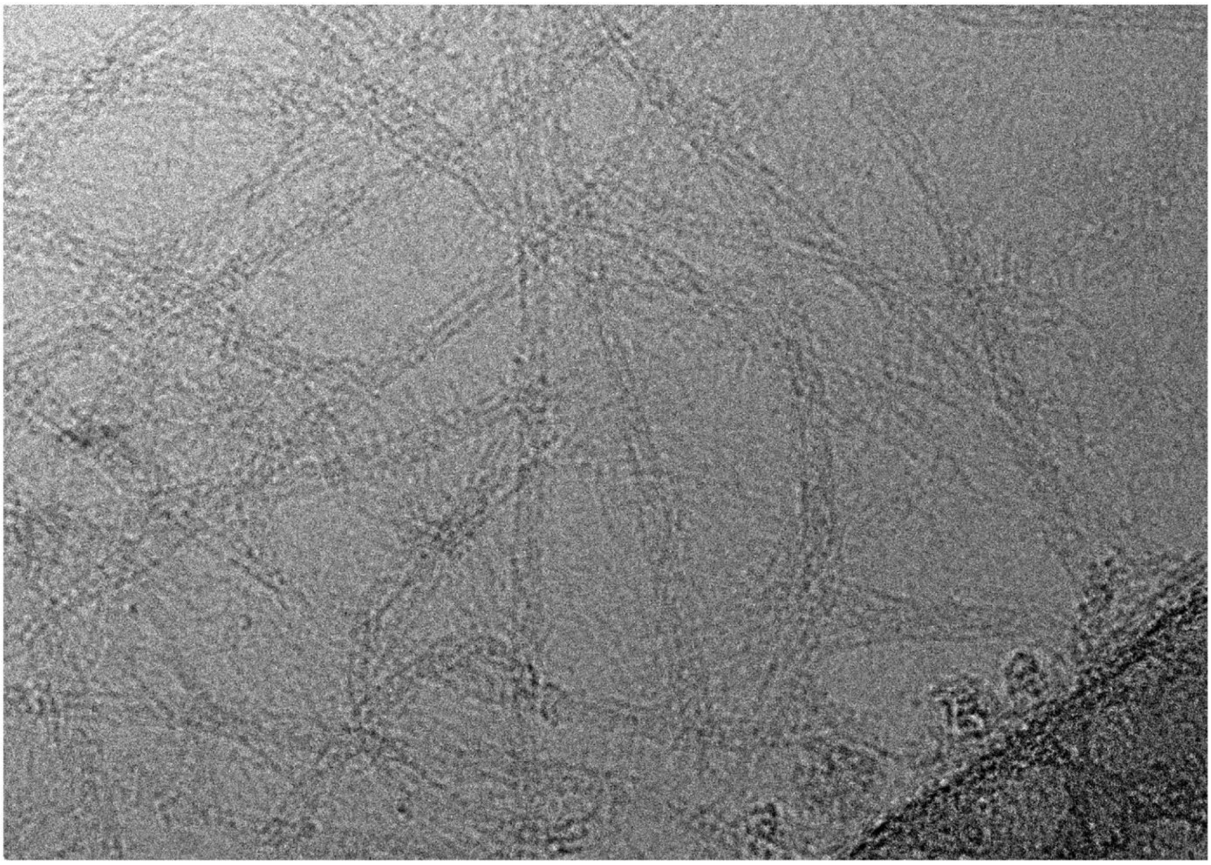

Supplementary Figure 4. Micrograph of in-vitro polymerized human VIFs. Of this sample 12,160 micrographs were recorded. The field of view is 392 x 278 nm.

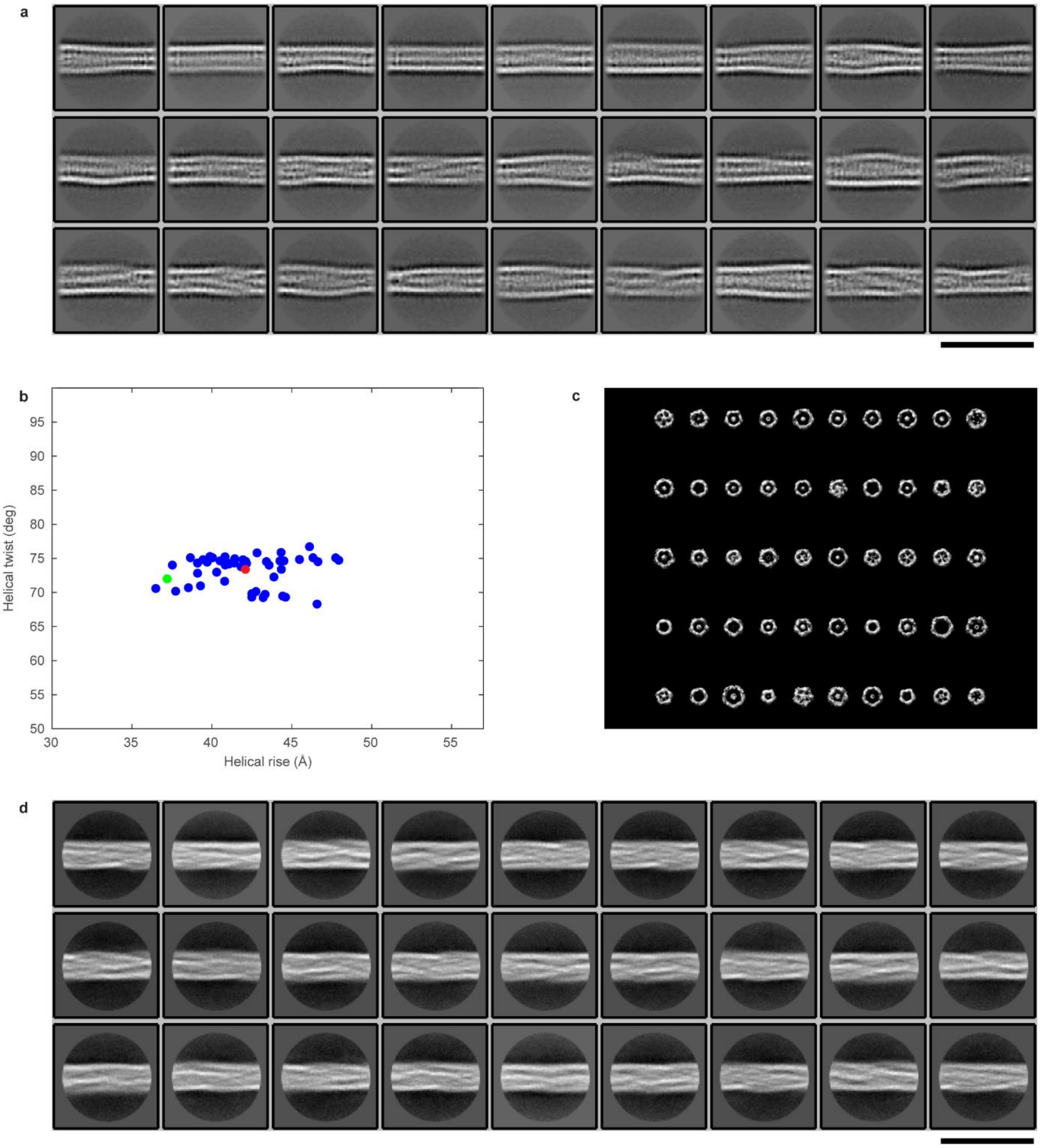

Supplementary Figure 5. Cryo-EM data processing. (**a**) Before sorting the VIF segments with extensive 3D classifications, their 2D classes showed the previously detected pattern, namely that one filament wall appears more pronounced in projection than its counterpart. However, at this stage of data processing the VIF segments were considerably heterogenous regarding their diameter. The image shows a representative subset of 27 out of 200 class averages calculated from 801,585 VIF segments. Scale bar is 35 nm. (**b**) The helical symmetry search based on the cryo-EM data was performed in an interval between 50° to 100° for the

helical twist and 30 Å to 57 Å for the helical rise, therefore also capturing possible helical assemblies based on 4 or 6 protofibrils. As starting value for the symmetry search an initial twist angle of 72° and an initial helical rise of 37 Å was set (green dot), and this helical symmetry was also imposed on the initial 3D template. In between the helical 3D classifications, those 3D classes converging to the borders of the search interval were iteratively removed. In the final helical 3D classification (the resulting helical symmetry parameter are shown as blue dots in the plot and the corresponding class averages are shown in (**c**)), the mean helical symmetry was 73.4° for the helical twist angle and 42.1 Å for the helical rise (red dot). This helical symmetry parameter were used as the initial twist and rise values for subsequent local helical symmetry searches during 3D refinement, finally converging to the exact identical helical symmetry parameter as determined before based on power spectrum analysis. (**d**) The VIF segments underlying the final VIF 3D structure were combined with 2D classification to assess the gain of structural homogeneity during data processing. The image shows a representative subset of 27 out of 100 class averages calculated from the final 236,920 VIF segments. Scale bar is 35 nm.

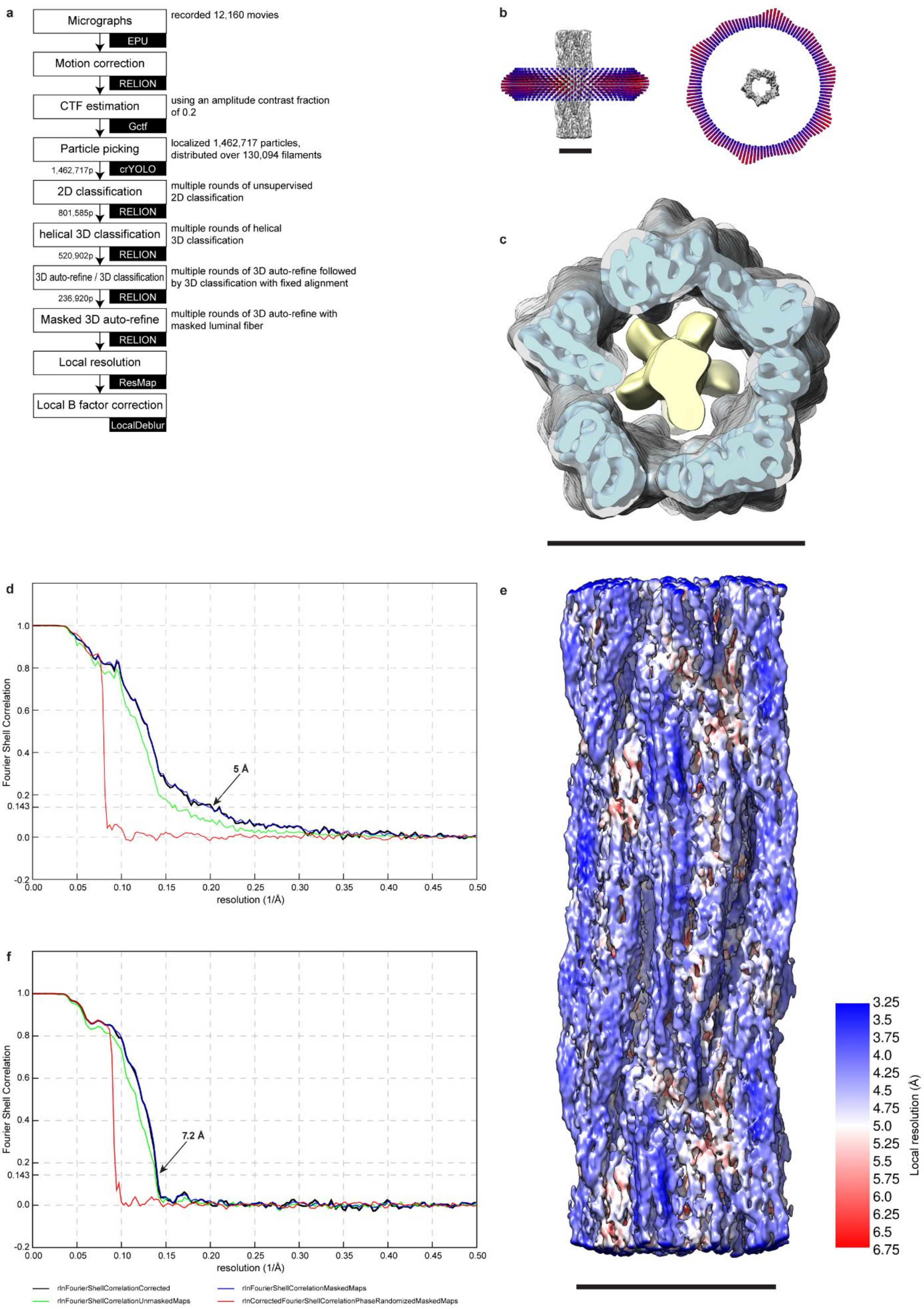

Supplementary Figure 6. Cryo-EM data processing workflow and resolution measurement. (**a**) Single particle processing workflow. See Methods section for more details.

(**b**) Visualization of the angular distribution of the final VIF 3D structure (Fig. 2). (**c**) In order to reach subnanometer resolution it was critical to mask the luminal fiber during 3D refinement. The blue colored density shows the final VIF average and the grey density the mask applied during the final 3D refinement. The structure of the luminal fiber (yellow density) was obtained from a preceding refinement (resolution ~14 Å) without masking the luminal fiber. For visualization the final VIF structure was combined with the luminal fiber structure (Fig. 2). (**d**) Calculating the gold-standard Fourier shell correlation (FSC) curve with the RELION command relion_postprocess, and using the option --ampl_corr, indicates a global resolution of 5.0 Å at FSC = 0.143. (**e**) Local resolution measurement of the electron density map. (**f**) The gold-standard FSC curve of the final VIF 3D structure indicates an overall resolution of 7.2 Å at FSC = 0.143.

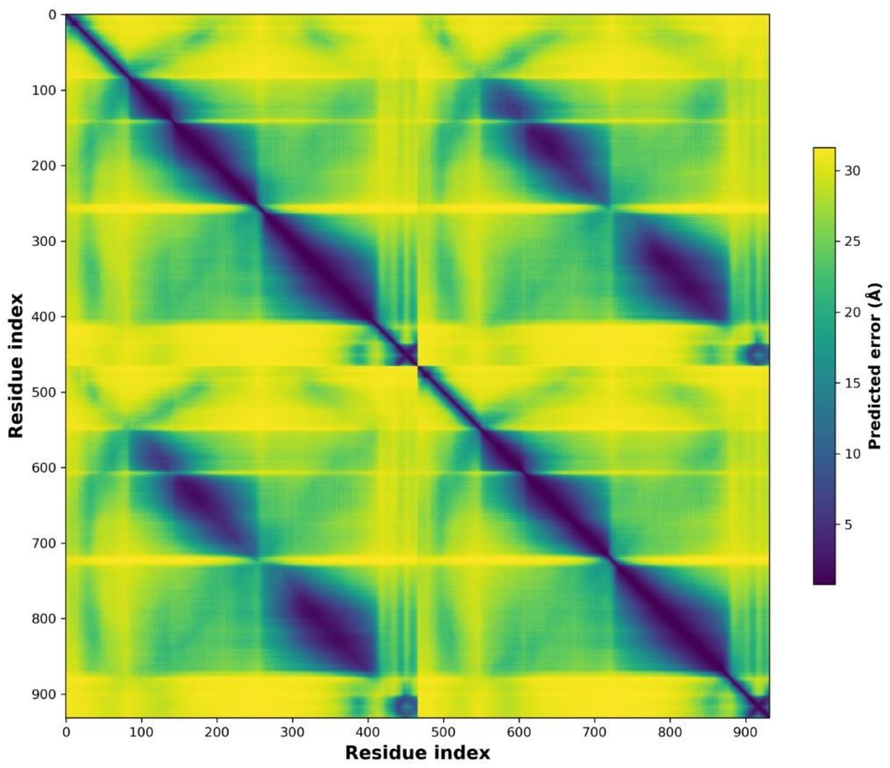

Supplementary Figure 7. Alphafold predicted aligned error (PAE) plot of the initial vimentin dimer model.

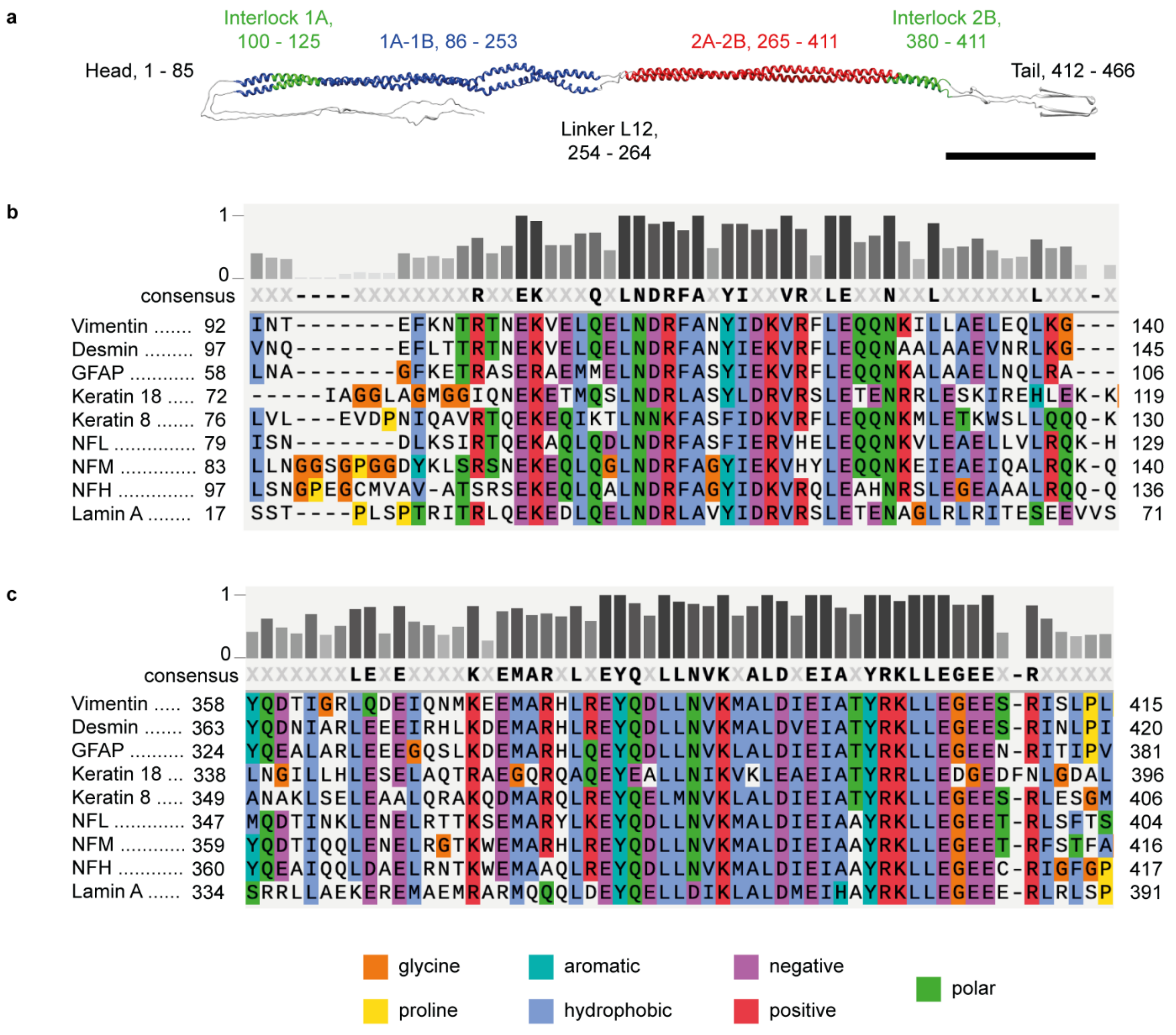

Supplementary Figure 8. Sequence conservation of the interlock region between different IF proteins. (**a**) Model of the vimentin dimer (1A-1B domains blue, 2A-2B domains red. The two interlock regions in coil 1A and coil 2B are colored green. The head and tail domains, as well as linker L12 are displayed in grey. Scale bar is 10 nm. (**b,c**) Multiple sequence alignment of human isoforms of vimentin (UniProt accession code P08670), desmin (P17661) and the glial fibrillary acidic protein, GFAP (P14136) (type III IFs), keratin 18 (P05783) and 8 (P05787) (type I and II), neurofilaments light, medium and heavy chains, NFL (P07196), NFM (P07197), NFH (P12036) (type IV) and lamin A (P02545) (type V). The grayscale histogram shows the relative sequence conservation, where 1 indicates the maximum score. The consensus sequence is shown with a threshold of >70%.

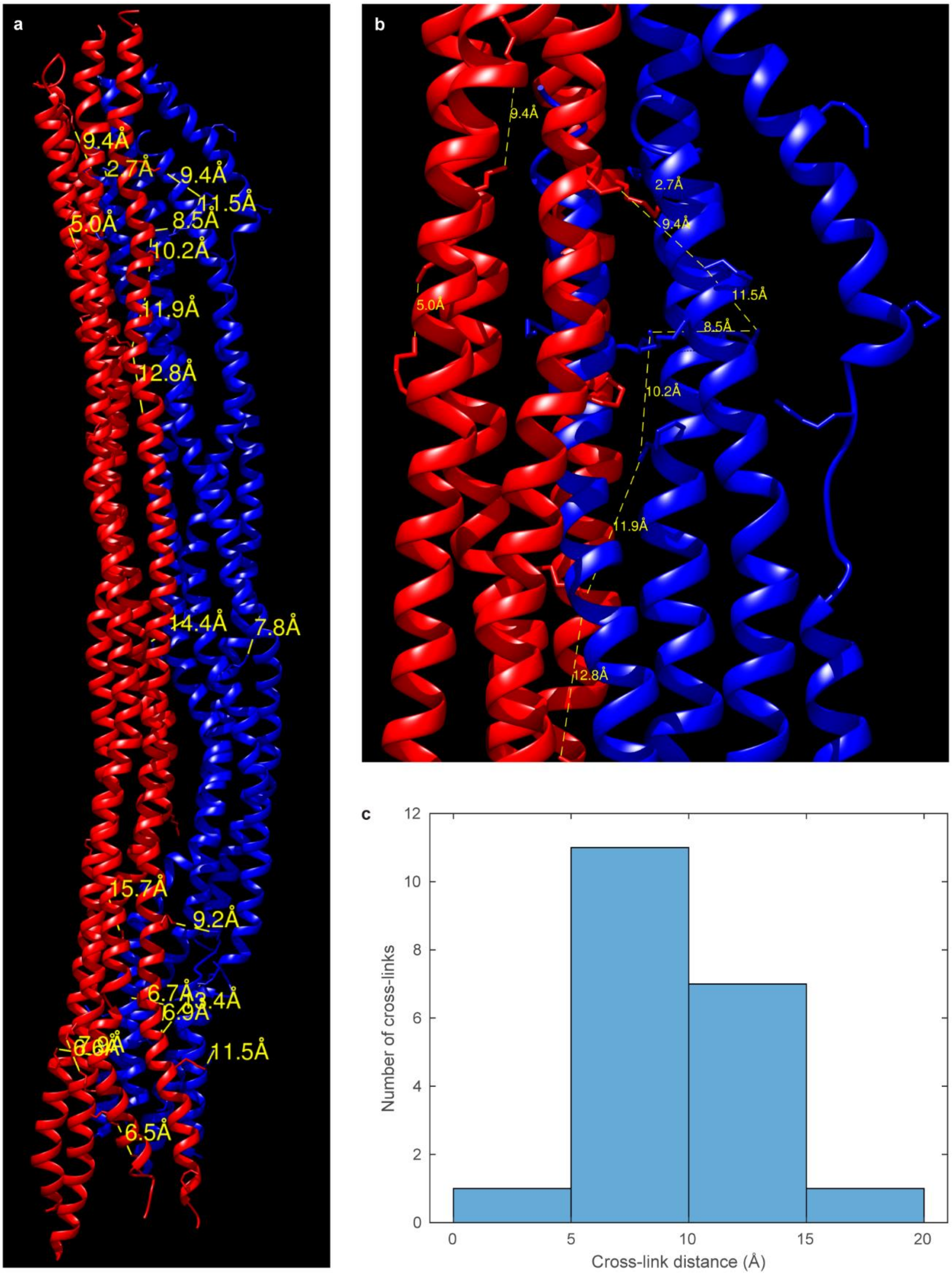

Supplementary Figure 9. Mapping of cross-links. (**a**) Previously reported cross-links (yellow dashed lines) were superpositioned onto a model of the protofibril repeating unit (2A-2B dimers colored red, 1A-1B dimers colored blue). The cross-links were taken from references [2, 3]. (**b**) Zoom into the interlock region. (**c**) Histogram of the distances between the cross-linked residues in the model. The mean distance is 9.4 Å.

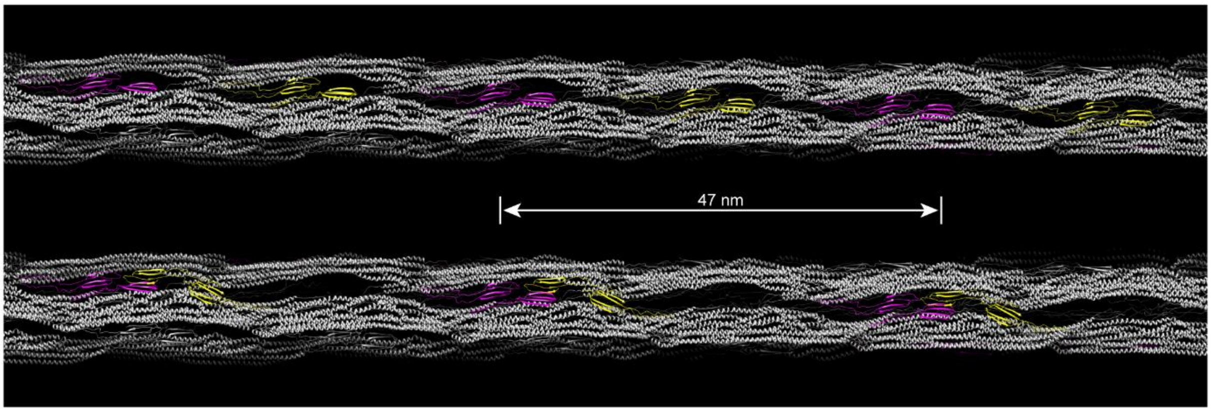

Supplementary Figure 10. Tail clustering model. In the VIF model, the tail domains are evenly distributed along the filament (upper model, tail domains are colored alternating between magenta and yellow). If the tail domains reorganize and would form clusters along the VIFs (lower model), the distance between these tail domain clusters would be ~47 nm. This could explain previous measurements of the distance between the tail domains [4, 5].

References

1. Martins, B., et al., *Unveiling the polarity of actin filaments by cryo-electron tomography.* Structure, 2021.
2. Steinert, P.M., L.N. Marekov, and D.A. Parry, *Diversity of intermediate filament structure. Evidence that the alignment of coiled-coil molecules in vimentin is different from that in keratin intermediate filaments.* J Biol Chem, 1993. **268**(33): p. 24916-25.
3. Parry, D.A., L.N. Marekov, and P.M. Steinert, *Subfilamentous protofibril structures in fibrous proteins: cross-linking evidence for protofibrils in intermediate filaments.* J Biol Chem, 2001. **276**(42): p. 39253-8.
4. Lin, Y., et al., *Toxic PR Poly-Dipeptides Encoded by the C9orf72 Repeat Expansion Target LC Domain Polymers.* Cell, 2016. **167**(3): p. 789-802 e12.
5. Nunes Vicente, F., et al., *Molecular organization and mechanics of single vimentin filaments revealed by super-resolution imaging.* Sci Adv, 2022. **8**(8): p. eabm2696.
