# Supplementary material for: Vimentin filaments integrate low-complexity domains in a complex helical structure
Source: Nat Struct Mol Biol. 2024 Apr 17;31(6):939–49. doi: 10.1038/s41594-024-01261-2 (PMC11189308; doi:10.1038/s41594-024-01261-2)
Supplement: Supplementary file 2 — Reporting Summary [file 41594_2024_1261_MOESM2_ESM.pdf]

nature portfolio

Corresponding author(s): Matthias Eibauer and Ohad Medalia

Last updated by author(s): Feb 14, 2024

Reporting Summary

Nature Portfolio wishes to improve the reproducibility of the work that we publish. This form provides structure for consistency and transparency in reporting. For further information on Nature Portfolio policies, see our Editorial Policies and the Editorial Policy Checklist.

Statistics

For all statistical analyses, confirm that the following items are present in the figure legend, table legend, main text, or Methods section.

n/a	Confirmed	
☐	☒	The exact sample size (*n*) for each experimental group/condition, given as a discrete number and unit of measurement
☐	☒	A statement on whether measurements were taken from distinct samples or whether the same sample was measured repeatedly
☒	☐	The statistical test(s) used AND whether they are one- or two-sided<br>*Only common tests should be described solely by name; describe more complex techniques in the Methods section.*
☒	☐	A description of all covariates tested
☒	☐	A description of any assumptions or corrections, such as tests of normality and adjustment for multiple comparisons
☐	☒	A full description of the statistical parameters including central tendency (e.g. means) or other basic estimates (e.g. regression coefficient) AND variation (e.g. standard deviation) or associated estimates of uncertainty (e.g. confidence intervals)
☒	☐	For null hypothesis testing, the test statistic (e.g. *F*, *t*, *r*) with confidence intervals, effect sizes, degrees of freedom and *P* value noted<br>*Give P values as exact values whenever suitable.*
☒	☐	For Bayesian analysis, information on the choice of priors and Markov chain Monte Carlo settings
☒	☐	For hierarchical and complex designs, identification of the appropriate level for tests and full reporting of outcomes
☒	☐	Estimates of effect sizes (e.g. Cohen's *d*, Pearson's *r*), indicating how they were calculated

Our web collection on statistics for biologists contains articles on many of the points above.

Software and code

Policy information about availability of computer code

Data collection	SerialEM (3.4.9, 3.8.0, 3.9.0), EPU (2.14)
Data analysis	MATLAB (R2019b)<br>TOM toolbox (1.0)<br>Actin Polarity Toolbox (APT, 1.0)<br>IMOD (4.7.15, 4.9.12)<br>IsoNet (0.1)<br>MotionCorr (2.1)<br>MotionCor2 (1.4.0)<br>Gctf (1.06)<br>crYOLO (1.8.2)<br>EMAN2 (1.9, 2.3)<br>RELION(3.0.8, 4.0.0)<br>UCSF Chimera (1.15)<br>Segger (2.5.3)<br>LocalDeblur (as implemented in scipion (3.0.11))<br>ResMap (1.95)<br>alphafold (2.1.2)<br>ClusPro (2.0)<br>AreTomo (1.3.3)<br>cryoSPARC (4.3)

Clustal Omega (1.2.2)
Nikon Elements (4.5)
Phenix (1.21)
Topaz (0.2.4)

For manuscripts utilizing custom algorithms or software that are central to the research but not yet described in published literature, software must be made available to editors and reviewers. We strongly encourage code deposition in a community repository (e.g. GitHub). See the Nature Portfolio guidelines for submitting code & software for further information.

Data

Policy information about availability of data

All manuscripts must include a data availability statement. This statement should provide the following information, where applicable:

- Accession codes, unique identifiers, or web links for publicly available datasets
- A description of any restrictions on data availability
- For clinical datasets or third party data, please ensure that the statement adheres to our policy

The VIF subtomogram average, the single particle VIF and single particle VIF-deltaT structures have been deposited in the Electron Microscopy Data Bank under the accession codes: EMD-19562, EMD-16844, EMD-19563. The VIF model have been deposited in the Protein Data Bank under the accession code PDB-8RVE. The integrative modelling protocol, initial alphafold models and the VIF tetramer model have been deposited in the PDBDEV under the accession code PDBDEV-00000212.
The UniProt code of human vimentin protein used for structure prediction with alphafold is P08670. For visualization of the complete cytoskeleton PDB models of F-actin (PDB-8A2R) and microtubules (PDB-6DPU) were used.

Human research participants

Policy information about studies involving human research participants and Sex and Gender in Research.

Reporting on sex and gender	N/A
Population characteristics	N/A
Recruitment	N/A
Ethics oversight	N/A

Note that full information on the approval of the study protocol must also be provided in the manuscript.

Field-specific reporting

Please select the one below that is the best fit for your research. If you are not sure, read the appropriate sections before making your selection.

☒ Life sciences ☐ Behavioural & social sciences ☐ Ecological, evolutionary & environmental sciences

For a reference copy of the document with all sections, see nature.com/documents/nr-reporting-summary-flat.pdf

Life sciences study design

All studies must disclose on these points even when the disclosure is negative.

Sample size	In total, 102 tomograms were acquired for the cryo-FIB/cryo-ET analysis from >3 different batches of cells. A subset of 7 tomograms was analyzed, which was sufficient to generate a subtomogram average showing the VIF protofibril stoichiometry.<br>In total, 225 tomograms were acquired of detergent-treated MEFs from >3 different batches of cells, which was sufficient to determine the helical parameters of VIFs.<br>For the VIF single particle structure 12,160 micrographs were recorded, which was sufficient to generate a subnanometer resolution structure. For the VIF-deltaT single particle structure 19,534 micrographs were recorded, which was sufficient to generate a structure which showed the position of the tail domains.
Data exclusions	Individual VIF subtomograms or single particles, which did not contribute to an improvement of resolution in subtomogram averaging or single particle analysis were excluded based on unsupervised 2D classification and 3D classification.
Replication	At least 3 independent, successful replications were performed for all EM measurements.
Randomization	For subtomogram averaging and single particle analysis the 3D refinement and averaging procedures were performed with random individual half sets.
Blinding	Given the fact that the structure of VIFs was unknown to any researcher involved in this study, blinding was not relevant.

Reporting for specific materials, systems and methods

We require information from authors about some types of materials, experimental systems and methods used in many studies. Here, indicate whether each material, system or method listed is relevant to your study. If you are not sure if a list item applies to your research, read the appropriate section before selecting a response.

Materials & experimental systems

n/a	Involved in the study
☐	☒ Antibodies
☐	☒ Eukaryotic cell lines
☒	☐ Palaeontology and archaeology
☒	☐ Animals and other organisms
☒	☐ Clinical data
☒	☐ Dual use research of concern

Methods

n/a	Involved in the study
☒	☐ ChIP-seq
☒	☐ Flow cytometry
☒	☐ MRI-based neuroimaging

Antibodies

Antibodies used

- anti-vimentin antibody (dilution 1:200, catalog number 919101, Biolegend, USA)
- lamin A/C antibody (dilution 1:100, catalog number sc-376248, Santa Cruz Biotechnology, USA)
- anti-chicken secondary antibody (dilution 1:400, catalog number A-11039, Invitrogen, USA)
- anti-rabbit secondary antibody (dilution 1:400, catalog number A-11011, Invitrogen, USA)

Validation

- Validation for anti-vimentin antibody: https://www.biolegend.com/de-de/products/purified-anti-vimentin-antibody-11598
- Validation for lamin A/C antibody: https://www.scbt.com/p/lamin-a-c-antibody-e-1?gad_source=1&gclid=EAIaIQobChMI8N7noNiqhAMVboVoCR1qQwRwEAAYASAAEgIz2vD_BwE
- Validation for anti-chicken secondary antibody: https://www.thermofisher.com/antibody/product/Goat-anti-Chicken-IgY-H-L-Secondary-Antibody-Polyclonal/A-11039
- Validation for anti-rabbit secondary antibody: https://www.thermofisher.com/antibody/product/Goat-anti-Rabbit-IgG-H-L-Cross-Adsorbed-Secondary-Antibody-Polyclonal/A-11011

Eukaryotic cell lines

Policy information about cell lines and Sex and Gender in Research

Cell line source(s)

The MEF cell line was received from the Eriksson lab, Åbo Akademi University, Turku, Finland.

Authentication

The MEF cell line was authenticated in Virtakoivu et al. Cancer Res (2015) 75 (11): 2349–2362 (https://doi.org/10.1158/0008-5472.CAN-14-2842).

Mycoplasma contamination

The MEF cell line tested negative for mycoplasma contamination.

Commonly misidentified lines
(See ICLAC register)

No commonly misidentified cell lines were used in this study.
